# The Utilities

**Published:** 1881-11

**Authors:** 


### THE UTILITIES.

**Eating** and Drinking. The French are more dainty than we are. We saw an artist one day in the Louvre, after being intently occupied in making a copy of a beautiful painting, lay down his easel, retire behind a column, pull a bottle of wine out of one pocket and a loaf of bread out of the other,and dine upon them with as much apparent satisfaction as an American at the most expensive dinner. Statistics show that each person in Paris, on an average, consumes in one year thirty-two gallons of wine and one hundred and sixty-four pounds of meat. A laboring man in America consumes about six pounds of food and drink every day.

Many persons bring on life-long dyspeptias by the mere habit of drinking several glasses of cold water at their meals, an equal amount of hot drink would be greatly more advantageous, but half a pint of any fluid at a single meal is abundant for all healthful purposes. There is very little real nutriment in the beverages taken at meals. There is as much nourishment in a pound loaf of bread as there is in one hundred and forty-six gallons of the best beer, and yet beer and ale and porter are constantly recommended to invalids as nourishing, porter be it remembered being nothing more than unsaleable beer colored with burnt malt to hide the defects. But even as to beer, if the fermentation is complete, there is not an atom of nourishment in a barrel of it ; it is only bad or imperfect beer that has even a trace of nutriment. It is true that beer is made out of grain, but the grain must be thoroughly rotted before it yields beer ; hence it is that beer gives no real strength, the alcohol in it gives apparent strength for a time, but soon the same amount of stimulus ceases to stimulate ; meanwhile they wake up to the fact and express it in this wise, "my friends say I look better and am fleshing up,but somehow or other I don't gain strength," and very soon after, in multitudes of cases, the system, to use a sailor's expression, "goes down with a run."

ANIMAL FOOD.—Different nations instinctively fall into the habit of using the kind of food adapted to their latitude, habits and localities; The Frenchman luxuriates on bread and wine in his sunny clime : the Englishman in everlasting fog and dreariness, leans heavily on beef and beer ; the Dutch delight in sour krout and sausage ; pork and beans, clams and pumpkin pies always delight the lean Yankee ; while Western men know no heaven where there is not hog and homminy ; John Chinaman makes rice the god of his idolatry ; Italians feast the year round on maccaroni ; the Cuban is happy amid his plantain trees, while Greenlanders believe in blubber as the summum bonum of human good ; what would a Paddy be without his potatoe, or Sandy without his luxurious oat meal ; and his hunting ground is the heaven of the Indian. The cannibals of a thousand years ago, are the same lovers of human flesh to-day : and none of these nations have ever died out, all of them seem to live and thrive on the aliment which a munificent Providence has strewn so abundantly around them. The fishermen of the Ferroe Isles live mainly on the yield of their nets, as their fathers did before them, and are the healthiest people on the globe. These facts seem to show the absurdity of the vagaries of many who set themselves up as reformers and would be saviors of the race, closing their eyes against the glaring fact that the food of the individual must be adapted to his temperament, his locality and his occupation. But in all this, the great truth stands out with unmistakable prominence that God is good, in that intending man to habitate the globe he has adapted him, with reasonable restrictions to live any where and on any thing. And while witless hosts are ranging themselves in hostile fronts as meaters and anti-meaters, vegetarians and grapeites, (for a book has been really written to prove that to live long and healthfully we must eat grapes all day), sensible people will eat in moderation what they like best according to nature's instincts, taking their food in moderation, taking care that the fruits should be ripe and perfect, the vegetables fresh, the meat taken from well fed and healthy carcasses and all cleanlily prepared, thoroughly cooked, served in simple style, and eaten in contentment, thankfulness and joviality. Paddy at home seldom smells meat except at Christmas and Easter ; when he comes to America he eats meat three times a day, and, if he lets liquor alone, accumulates money and becomes a steady, useful citizen. After seventeen years of observation. the overseer of the Devon Estates in Ireland makes the suggestive statement : " there are 6,680 persons on the estate. They are energetic, moral and well behaved. I do not remember a crime in seventeen years, not even so much as stealing a chicken. They are a contented, grateful people—grateful even for fair play. Out of six hundred farmers, deduct fifty, and the rest do not see a wheaten loaf, or smell meat, except at Christmas and Easter. They have been brought up to this custom. One tenant on the Devon estate I have seen sit down to potatoes, buttermilk and Indian meal who purchased at a recent sale $50,000 worth of property, and did not have to borrow a shilling to pay for it.

